# Calcific aortic valve disease augments vesicular microRNA-145-5p to regulate the calcification of valvular interstitial cells via cellular crosstalk

**DOI:** 10.1007/s00395-025-01133-w

**Published:** 2025-08-22

**Authors:** P. R. Goody, D. Christmann, D. Goody, S. Hildebrand, H. Billig, D. Nehl, R. Chennupati, M. Gladka, K. Wilhelm-Jüngling, S. Uchida, S. Iris-Bibli, J. B. Moore, N. Hamdani, F. Paneni, S. S. Pullamsetti, S. Zimmer, F. Jansen, F. Bakhtiary, E. Aikawa, A. Pfeifer, G. Nickenig, M. R. Hosen

**Affiliations:** 1 Heart Center Bonn, Molecular Cardiology, Department of Internal Medicine II, University Hospital Bonn, Venusberg-Campus 1, 53127 Bonn, Germany; 2 Institute of Pharmacology and Toxicology, University Hospital Bonn, Venusberg-Campus 1, 53127 Bonn, Germany; 3 Department of Cardiology, Pulmonology and Vascular Medicine, Medical Faculty and University Hospital, Heinrich-Heine University, 40225 Düsseldorf, Germany; 4 Department of Medical Biology, Amsterdam University Medical Centers, Amsterdam Cardiovascular Sciences, Meibergdreef 15, 1105 AZ Amsterdam, The Netherlands; 5 Endothelial Signaling and Metabolism, Institute for Cardiovascular Sciences, University Hospital Bonn, Venusberg-Campus 1, 53127 Bonn, Germany; 6 Center For RNA Medicine, Department of Clinical Medicine, Aalborg University, Frederikskaj 10B, 2, 2450 Copenhagen SV, Denmark; 7 Department of Vascular Dysfunction, European Center for Angioscinece (ECAS), Medical Faculty Mannheim, Heidelberg University, Ludolf-Krehl-Straße 13-17, 68167 Mannheim, Germany; 8 Center for Cardiometabolic Science, Department of Medicine, University of Louisville, 580 S. Preston Street, Louisville, KY 40202 USA; 9 Department of Cellular and Translational Physiology, Faculty of Medicine, Institute of Physiology, Universitätsstr. 150, 44801 Bochum, Germany; 10 Center for Translational and Experimental Cardiology (CTEC), Department of Cardiology, University Hospital Zurich and University of Zürich, Wagistrasse 12, 8952 Schlieren, Switzerland; 11 Lung Vascular Epigenetics, Max-Planck-Institute for Heart and Lung Research, Parkstr. 1, 61231 Bad Nauheim, Germany; 12 Department of Cardiac Surgery, Heart Center Bonn, University Hospital Bonn, Venusberg-Campus 1, 53127 Bonn, Germany; 13 Division of Cardiovascular Medicine, Brigham and Women’s Hospital, Harvard Medical School, 77 Ave Louis Pasteur, NRB 741, Boston, MA 02115 USA

**Keywords:** Aortic valve stenosis, MicroRNA, Extracellular vesicles, Cellular crosstalk, Valvular calcification

## Abstract

Calcific aortic valve disease (CAVD) is one of the leading causes of cardiovascular death in the elderly population worldwide. MicroRNAs (miRNAs) are highly dysregulated in patients with CAVD undergoing surgical aortic valve replacement (SAVR). However, the miRNA-dependent mechanisms regulating inflammation and calcification or miRNA-mediated cell–cell crosstalk during the pathogenesis of CAVD remain poorly understood. Here, we investigated the role of extracellular vesicle (EV)-associated *miR-145-5p*, which we showed to be strongly upregulated in CAVD in mice and humans during valve calcification. Human TaqMan miRNA arrays identified dysregulated miRNAs in CAVD tissue explants from patients compared to non-calcified (patients with aortic insufficiency) heart valve tissue explants from patients undergoing SAVR. Echocardiographic parameters were measured in conjunction with the quantification of dysregulated miRNAs in a murine CAVD model. In vitro calcification experiments were performed to investigate the effects of EV-*miR-145-5p* on calcification and crosstalk in heart valve cells. Integrated OMICS analyses were performed to analyze molecular miRNA signatures and their effects on signaling pathways-associated with CAVD. RNA sequencing, high-throughput transcription factor (TF) activity assays, and osteogenesis arrays revealed that a number of genes, miRNAs, TFs are critical for calcification and apoptosis involved in the pathogenesis of CAVD. Among several miRNAs dysregulated in valve explants from CAVD patients, *miR-145-5p* was the most highly sex-independently upregulated miRNA (AUC, 0.780, *p *value, 0.01) in patient plasma. Large EV population (170–800 nm) isolated from aortic valve tissues explanted from patients with CAVS (calcific aortic valve stenosis) after SAVR demonstrated a significantly higher level of *miR-145-5p* expression in comparison to control (vesicle-free plasma). MiRNA arrays utilizing with aortic stenosis samples from patients and mice showed that the expression of *miR-145-5p* is significantly upregulated and positively correlated with cardiac function based on echocardiography. In vitro experiments confirmed that *miR-145-5p* is encapsulated in EVs and transported into interstitial cells of the aortic valve. The results of integrated OMICs show that *miR-145-5p* is related to markers of inflammation, calcification, and apoptosis. In vitro calcification experiments demonstrated that *miR-145-5p* regulates the *ALPL* gene, a hallmark of calcification in vascular and heart valve cells. Mechanistically, EV-mediated shuttling of *miR-145-5p* suppressed the expression of *ZEB2*, a negative regulator of the *ALPL* gene, by binding to its 3' untranslated region to inhibit its translation, thereby diminishing the calcification of valvular interstitial cells. Elevated levels of pro-calcific and pro-apoptotic EV-associated *miR-145-5p* contribute to the progression of CAVD via the *ZEB2-ALPL* axis, which could potentially be therapeutically targeted to minimize the burden of CAVD.

**Graphical abstract:**

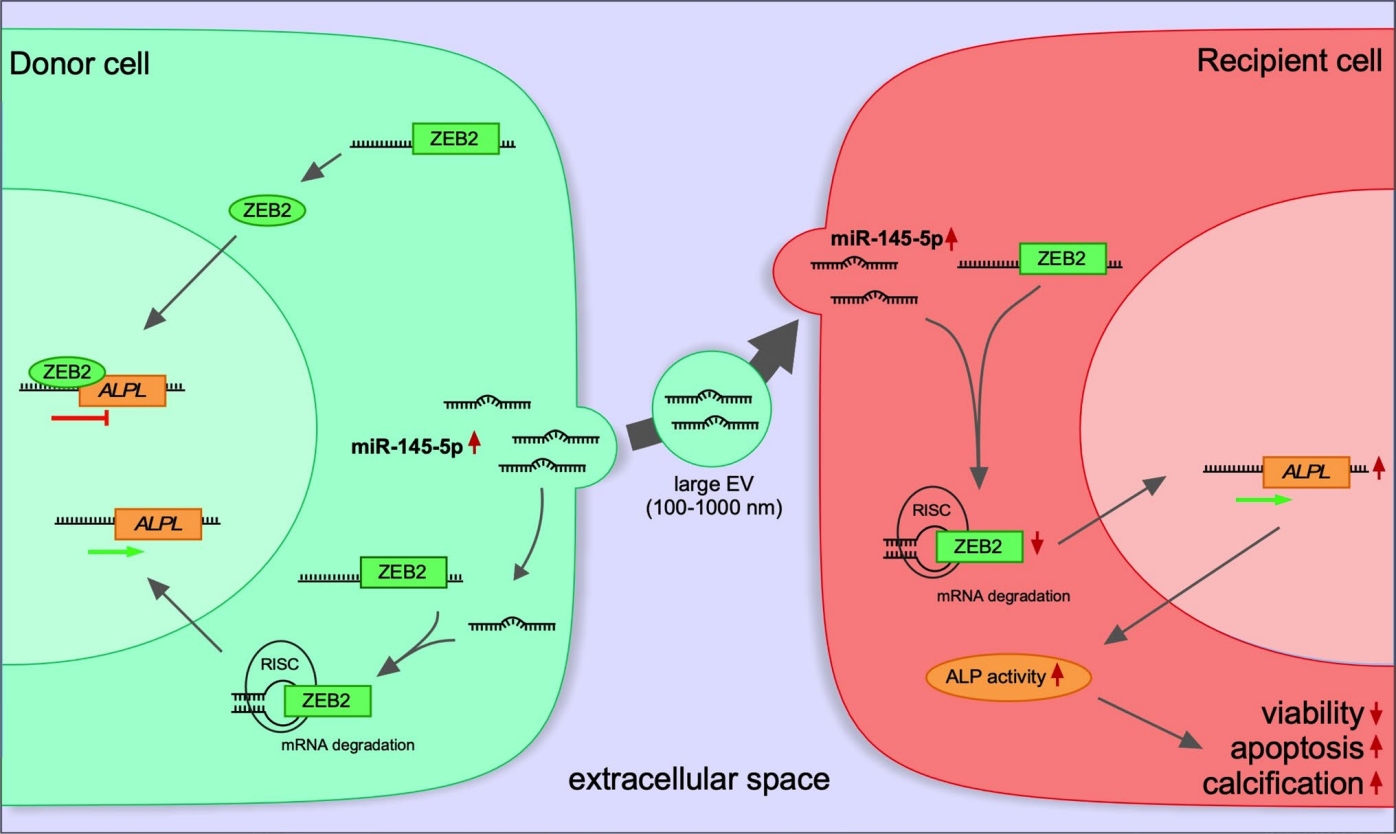

**Supplementary Information:**

The online version contains supplementary material available at 10.1007/s00395-025-01133-w.

## Introduction

Aortic valve (AV) disease is a significant contributor to cardiovascular death worldwide and shows a prevalence of over 2% in cardiovascular patients over 60 years of age [28]. The 2-year mortality rate is greater than 50% when symptoms of severe calcific aortic valve disease (CAVD) manifest, including dyspnea, angina pectoris, or cardiac syncope [27]. While CAVD is in part a degenerative disease, with “wear and tear”, chronic inflammation is also a significant contributor to disease pathogenesis driving pathological fibrosis and calcification of the valve cusps [27, 28]. CAVD can be divided into distinct phases. Initial endothelial damage, due to mechanical and shear stress, leads to infiltration of phospholipids (PL) and low-density lipoprotein particles (LDL). PL and LDL can be oxidized in the valve cusps, creating a pro-inflammatory milieu. Infiltration by monocytes and T cells characterizes the initiation phase of the disease. Pro-inflammatory cytokines, secreted by classically activated macrophages and CD-8^+^ T cells can induce apoptosis and differentiation of valvular interstitial cells (VIC) into osteoblast-like cells [23, 24]. Arising cell debris acts as a further promotor of inflammation and can function as an initiator for microcalcification. Increasing osteoblastic differentiation and forming macrocalcification are indicative of the next phase of CAVD, termed the propagation phase that culminates in formation of macrocalcification, characteristics of the advance stage of disease. Currently, no pharmacological treatments targeting either phase of the disease are available, and the only treatment option is surgical or interventional valve replacement (SAVR or TAVR) [9, 29, 40].

MicroRNAs (miRNAs) are small noncoding RNAs that are involved in cardiovascular diseases [9, 12, 13]. When compared to healthy valves, miRNAs have been shown to be differentially expressed in aortic valve tissues from patients undergoing valve replacement surgery due to CAVD [24]. MiRNAs can also be packaged into extracellular vesicles (EV) to be transferred between different cells [1, 2, 14]. This phenomenon has been investigated in many diseases, including different types of cancer and in atherosclerosis [2, 10, 11]. We and others have shown that miRNA transfer among endothelial cells (EC), cardiomyocytes (CM), and smooth muscle cells (SMC) induces direct genetic and phenotypic changes in the target cells [7, 25]. Recently, we showed that an increase in EV-*miR-122-5p* in patients with CAVD represents a novel mechanism for the deterioration of cardiac function in patients following TAVR [14]. EVs carry miR-122-5p and facilitate its transfer into cardiomyocytes (CM) through direct interaction with the multifunctional RNA-binding protein (RBP) heterogeneous nuclear ribonucleoprotein U (hnRNPU), thereby regulating CM viability [14]. Furthermore, vesicular shuttling of *miR-30c-5p* is regulated by hnRNPU in a sequence-specific manner, which controls EC function and is augmented in coronary artery disease [42]. EVs have also been shown to play a role during CAVD, with released EVs from VICs and macrophages acting as nucleation sites for microcalcification [33–36]. However, the horizontal transfer of vesicle-bound contents (e.g., miRNAs, proteins) and their effects on valvular calcification have not been well investigated.

Our study aims to explore whether tissue-resident miRNA contents differ between patients with and without CAVD. Further, we examined whether vesicular RNA contents are distinctive and how such differences impact disease progression through cell–cell communication via EVs. We demonstrated for the first time that vesicular *miR-145-5p* is increased in clinical and experimental settings of valvular calcification (i.e., in vitro calcification cell culture experiments, in patients with CAVD, and a murine model of CAVD). Further functional and mechanistic studies revealed that EV-*miR-145-5p* is an important regulator of valvular calcification and osteoblastic differentiation of intestinal cells via vesicular shuttling.

## Methods

A detailed methods section is provided in the online Data Supplement.

### Study approval and human specimen

All clinical samples and measurements were obtained after informed consent from patients following ethical approval by the ethics committee of the University of Bonn (approval number: AZ78/17). Aortic valve specimens were collected from patients undergoing SAVR for either severe aortic stenosis or aortic regurgitation in cooperation with the Department of Heart Surgery, University Hospital Bonn, Germany.

### Data availability

MiRNA array data are available from the Gene Expression Omnibus (GEO) under the accession number: GSE1905689. RNA-seq data are available from the GEO under the accession number GSE190539. The raw data of transcription factor arrays are provided as online data supplements. All further data that support the findings of this manuscript are available upon request from the corresponding author.

### Statistical analysis

Normally, distributed continuous variables were presented as the mean ± standard deviation (SD). Continuous variables were tested for normal distribution using the Kolmogorov–Smirnov test. Categorical variables are given as frequencies and percentages. For continuous variables, two-tail, unpaired Student’s t, and Mann–Whitney U tests were used for the comparison between the two groups. For the comparison of > 2 groups, the one-way ANOVA with Bonferroni correction for multiple comparisons test was used. All tests were two-sided. Statistical significance was assumed when the null hypothesis could be rejected at *p* < 0.05. Statistical analysis was performed with IBM SPSS Statistics version 20 (IBM Incorporation, USA) and GraphPad Prism 9 (GraphPad Inc, USA).

## Results

### Baseline characteristics and identification of differentially regulated miRNAs in valve tissue from CAVD patients post-SAVR

**Fig. 1:**
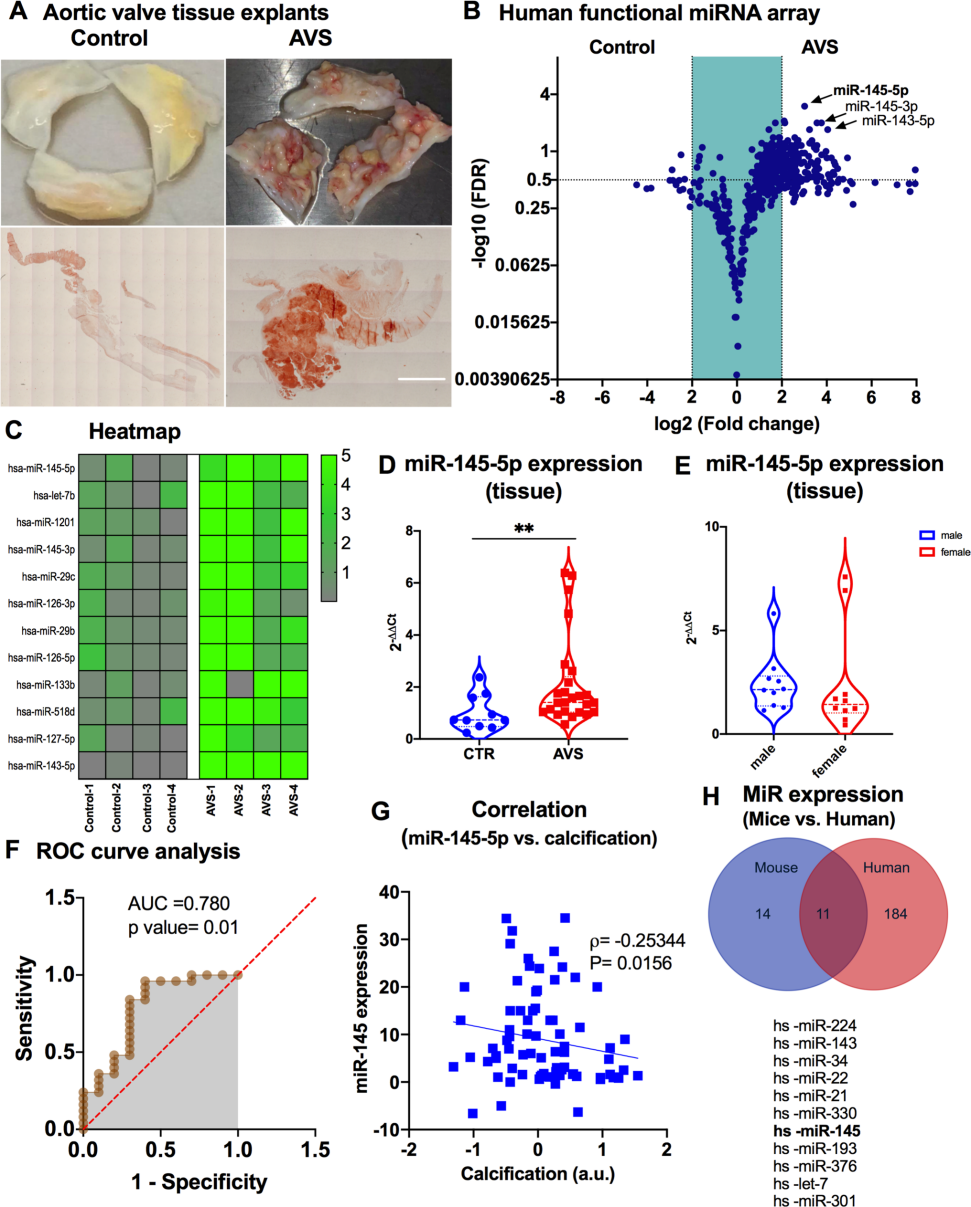
Investigation and profiling of miRNAs in stenotic aortic valves from patients who underwent surgical valve replacement with aortic valve stenosis. **A** Representative images of human calcified aortic valve (AVS) and non-calcified (no AVS = AI) aortic valves explanted from patients undergoing aortic valve replacement surgery (upper part) and stained with alizarin red staining (lower part). Scale bar = 2 µm. **B** Volcano plot showing differentially regulated human miRNAs in explanted valve tissues derived from patients who underwent SAVR. Thresholds (black dotted lines) of a two-fold change and *p *values (FDR-adjusted) < 0.05 were used to distinguish the miRNAs of interest. *n* = 4 for control (aortic insufficiency), *n* = 4 for aortic valve disease (AVS). **C** Heatmap showing top-regulated miR expression of tissue-resident miRs were analyzed in explanted aortic valve tissues derived from controls (*n* = 4) and AVS patients (*n* = 4). **D** Expression of tissue-associated *miR-145-5p* was analyzed in control and AVS patients by qRT-PCRs. Data represent the mean ± SEM (**p < 0.01, CTR, *n* = 10, AVS *n* = 30, by Student’s t test, two-tail, unpaired). **E** Expression of tissue-associated *miR-145-5p* was analyzed in male and female patients by qRT-PCRs. Data represent the mean ± SEM (ns, *p* < 0.06, Male, *n* = 12, AVS *n* = 12, by Student’s *t *test, two-tail, unpaired). **F** ROC curve analysis for the prediction of expression of *miR-145-5p* in patients with AVS and controls. (AUC, 0.780, **p value 0.01). **G** Correlation analysis of *miR-145-5p* expression with the level of calcification. (Spearman coefficient of *r* = − 0.25344, *p* = 0.0156). **H** Venn diagram representing the dysregulated and common miRNAs in aortic valve samples from mice (sham, *n* = 5, AVS, *n* = 5) and patients with AVS (control, *n* = 4, AVS, *n* = 4). Mice were subjected to a wire injury to induce aortic valve stenosis in vivo. Aortic valves from 5 mice were pooled and analyzed via miRNA array, and 5 aortic valves from sham-operated mice were used as controls. *MiR-145-5p* was found to be among the upregulated miRNAs in both human and murine stenotic valves. The common miRNAs are shown in the list with a cut-off > two-fold, *p *value < 0.05 (FDR-adjusted). SAVR, surgical aortic valve replacement; AVS, aortic valve stenosis; miRNA, microRNA; ROC, receiver operating characteristic curve

CAVD patients were characterized to identify differentially expressed miRNAs in valve tissues. Baseline characteristics showed that comorbidities affecting prognosis after SAVR [e.g., lung disease and coronary artery disease (CAD)] were similar in both groups (CAVS, calcific aortic valve stenosis; vs. AI, aortic insufficiency). Of note, other cardiovascular risk factors, such as type II diabetes (type II DM), body mass index (BMI), dyslipidemia, history of smoking, and creatinine levels, were elevated in patients with CAVD (Table 1). To identify differentially expressed miRNAs in valve tissue explants from CAVD patients, we performed an unbiased RT-qPCR-based human miRNA array in the screening cohort (Fig. 1A, B, Figure S1A). Explanted valves from patients with aortic regurgitation (Control, no CAVD) due to dilatation of the aortic root or ascending aorta served as controls when valve cusps did not exhibit signs of calcification. Explanted valves from CAVD patients showed calcifications, increased collagen deposition, elastin, and fibrin in aortic valve tissue sections stained with alizarin red and subsequently examined by light microscopy (Fig. 1A). Our array data showed that several miRNAs (*miR-145-5p, miR-let-7b, miR-1201, miR-145-3p, miR-29b, miR-126-3p, miR-29c, miR-126-5p, miR-133b, miR-518d, miR-127-5p, and miR-143-5p*) (Fig. 1B, C) were differentially expressed between CAVD patients and controls after SAVR [thresholds: |fold change|> 1.5 and p value < 0.05 (FDR-adjusted)]. Of these differentially expressed miRNAs, *miRNA-145-5p* showed a more than threefold (p = 0.001) increase in CAVD tissues compared to tissues from control patients (without CAVD). These results were recapitulated in a validation cohort using RT-qPCR, where *miR-145-5p* was significantly upregulated in CAVD patients compared to controls (*n* = 25 and *n* = 10, respectively; *p* = 0.01) (Fig. 1D). The increased expression of *miR-145-5p* was independent of gender with no significant difference observed between male and female CAVD patients in this validation cohort (Fig. 1E).

**Table 1:** Baseline characteristics of the study population

	Total (n = 54)	CAVD (n = 37)	Control (n = 17)	p value
Age (year)	65.2 ± 12	67.3 ± 10.1	60.7 ± 14.6	0,056
Weight (kg)	89.5 ± 19.2	92.4 ± 19.8	83.3 ± 16.5	0,117
Height (cm)	174.7 ± 9.8	174.5 ± 8.5	175.2 ± 13.1	0,819
Sex (no.;%)				0,675
Female	10(18.5%)	5 (13.5%)	5 (29.4%)	
Male	44 (81.5%)	32 (86.5%)	12 (70.6%)	
Cardiovascular risk factors				
Diabetes, n (%)	10(18.5%)	10 (27.0%)	0 (0%)	0,017
Dyslipidemia, n (%)	26 (48.1%)	21 (56.8%)	5 (29.4%)	0,064
CAD, n(%)	27 (50.0%)	20(54.1%)	7 (41.2%)	0,389
Hypertension, n (%)	34 (63.0%)	28 (75.7%)	13 (72.2%)	0,004
Smoker, n(%)	11 (20.4%)	9 (24.3%)	2 (11.8%)	0,296
PAD, n(%)	11 (20.4%)	9 (24.3%)	2 (11.8%)	0,296
LVEF	55.8 ± 9.6	56.2 ± 11.0	54.9 ± 5.8	0,57
BMI	26.9 ± 6.9	27.0 ± 4.7	25.3 ± 4.3	0,21
Atrial Fibrillation, n (%)	12 (22.2%)	4 (10.8%)	8 (47.1%)	0,002
Medical history				
Prior HF, n (%)	5 (7.4%)	4 (10.8%)	0 (0%)	0,165
Prior CKD, n (%)	12 (22.2%)	8 (21.6%)	4 (23.5%)	0,878
Prior Aortic aneurysm, n (%)	367 (13.4%)	0 (0%)	8 (47.1%)	0,067
Prior Stroke/TIA, n (%)	7 (13.0%)	3 (8.1%)	4 (23.5%)	0,122
Permanent Pacemaker	2 (3.7%)	2 (5.4%)	0 (0%)	0,338
Statin	30 (55.6%)	22 (59.5%)	8 (47.1%)	0,404
NOAK	11 (20.4%)	5 (13.5%)	6 (35.3%)	0,067
TAH	19 (35.2%)	15 (40.5%)	4 (23.5%)	0,232
Laboratory parameters				
CRP (mg/dl)	2.8 ± 3.4	2.9 ± 3.5	2.6 ± 3.1	0,734
eGFR (ml/min)	73.2 ± 14.8	73.6 ± 12.9	72.2 ± 18.8	0,742
Hb (g/dl)	14.2 ± 1.4	14.3 ± 1.3	13.9 ± 1.6	0,417
Leukocytes (G/l)	7.4 ± 2.5	7.6 ± 2.0	7.2 ± 3,4	0,566
Platelet count (G/l)	206.3 ± 55.4	203.0 ± 52.5	213.5 ± 62.3	0,551

Baseline demographic, laboratory and echocardiographic parameters of the validation study population. P values reflect the comparison between three different groups

*LVEF* left ventricular ejection fraction, *CAD* coronary artery disease, *HF* heart failure, *NOAK* novel oral anticoagulant drugs, *NYHA* New York heart association, *eGFR* estimated glomerular filtration rate, *TAH* total abdominal hysterectomy, *PAD* peripheral artery disease, *CKD* chronic kidney disease, *CRP* critical reaction protein, *BMI* body mass index, *Hb* hemoglobin, *AVA* aortic valve area

Among the differentially expressed miRNAs, only *miR-145-5p* showed significant diagnostic values in CAVD patients when ROC analysis was performed. This analysis suggested that *miR-145-5p* [CAVD vs. controls] was a reliable predictor of disease pathogenesis (AUC = 0.780, *p* = 0.01; Fig. 1F). We found that a significantly higher proportion of CAVD patients had increased *miR-145-5p* expressions. Furthermore, correlation analysis of *miR-145-5p* expression with the degree of calcification in CAVD and controls indicated that *miR-145-5p* is a critical regulator of the calcification status of CAVD patients (with a Spearman coefficient of *r* = − 0.25344, *p* = 0.0156) (Fig. 1G).

### MiR-145-5p is highly upregulated in both human stenotic aortic valves and the murine CAVD model

Most miRNAs are evolutionarily conserved, which facilitates understanding their functions using model animals such as mice. To search for species-conserved miRNAs in diseased aortic valve tissue, RT-qPCR-based miRNA arrays were performed using human (calcified tissue vs. control) and murine aortic valve tissue (wire injury vs. sham healing). Among the eleven miRNAs found to be differentially regulated (Fig. 1H), *miR-145-5p* was highly expressed in both humans and mice. Based on the above data, we aimed to investigate the functional significance and molecular mechanism of *miR-145-5p* in CAVD.

**Fig. 2:**
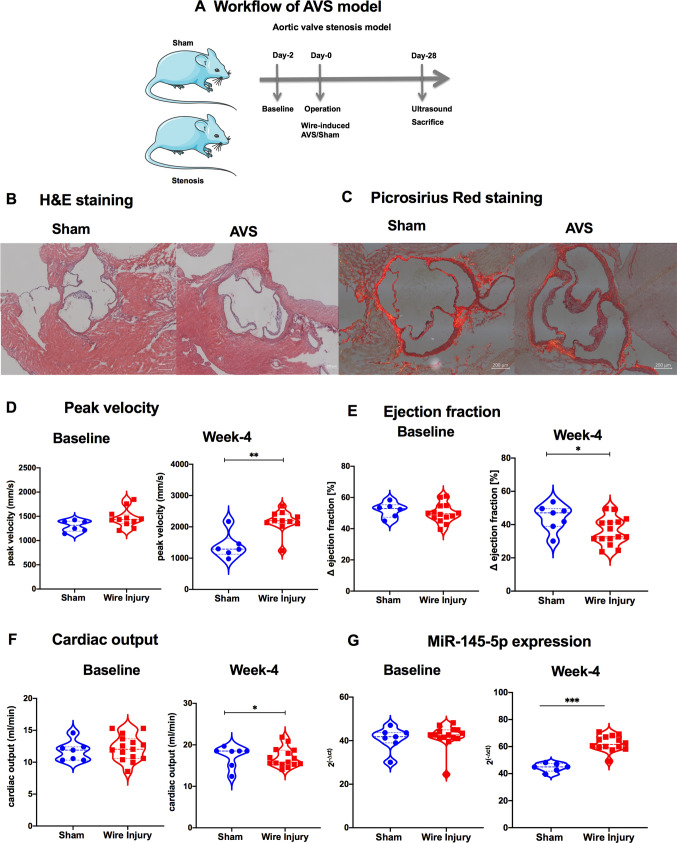
*MiR-145-5p* is differentially regulated in a murine model of aortic valve stenosis. **A** Timeline of the operation used to induce aortic valve stenosis in a murine model. The induction of aortic valve stenosis in mice is achieved by inserting a coronary wire into the left ventricle and rotating it. **B**, **C** Representative staining of hematolysin and eosin (H&E) and picosirius red for collagen. Change in morphology and collagen enrichments were observed in AV followed by AVS induction via graded wire. **D** Peak velocities over the aortic valve after sham operation or wire injury show induction of stenosis, as visualized with peak velocity, 4 weeks after surgery. Statistical significance was shown between, Sham-day 1 vs. Sham-week 4 and Stenosis-day 1 vs. Stenosis-week 4 (**p < 0.01, sham, *n* = 13; stenosis, *n* = 13; SEM; One-way ANOVA with Bonferroni multiple comparisons test). **E** Change in the ejection fraction after sham operation or operation creating severe stenosis on day 1 and week 4, as compared to before the surgery. Statistical significance was shown between, Sham-day 1 vs. Sham-week 4 and Stenosis-day 1 vs. Stenosis-week 4 (*p < 0.05, sham, *n* = 13; stenosis, *n* = 13; SEM; One-way ANOVA with Bonferroni multiple comparisons test). **F** Cardiac output after sham operation or operation creating severe stenosis on day 1 and week 4 (*p < 0.05, sham, *n* = 13; stenosis, *n* = 13; SEM; One-way ANOVA with Bonferroni multiple comparisons test). **G** *MiR-145-5p* expression, as determined by RT-qPCR, in valvular tissue after sham operation or operation creating severe stenosis on day 1 and week 4 (*p < 0.05, sham, *n* = 13; stenosis, *n* = 13; SEM; One-way ANOVA with Bonferroni multiple comparisons test). AVS, aortic valve stenosis

To further investigate the role of *miR-145-5p* in CAVD, we used a graded wire injury model of CAVD in mice (Fig. 2A) [21]. After sacrifice, hearts were isolated and sections were stained with hematoxylin and eosin to measure valve size (Fig. 2B). Picrosirius red staining was used to visualize collagen and fibrotic areas in sham and CAVD hearts, demonstrating an increase in collagen content and fibrotic area after wire injury (Fig. 2C). Wire-induced aortic valve injury led to the development of severe stenosis as evidenced by increased blood flow velocities four weeks after the surgery (Fig. 2D). Ejection fraction (EF) and cardiac output were inversely regulated in these mice, indicating that there is a CAVD-induced decrease of EF (Fig. 2E, F) compared to baseline (when compared to sham-operated mice). RT-qPCR analysis revealed that *miR-145-5p* was strongly upregulated in mice with CAVD (Fig. 2G), suggesting the involvement of miR-145-5p in pathogenesis of CAVD in mice.

### MiR-145-5p expression in interstitial cells is higher than in endothelial cells in valve tissues

**Fig. 3:**
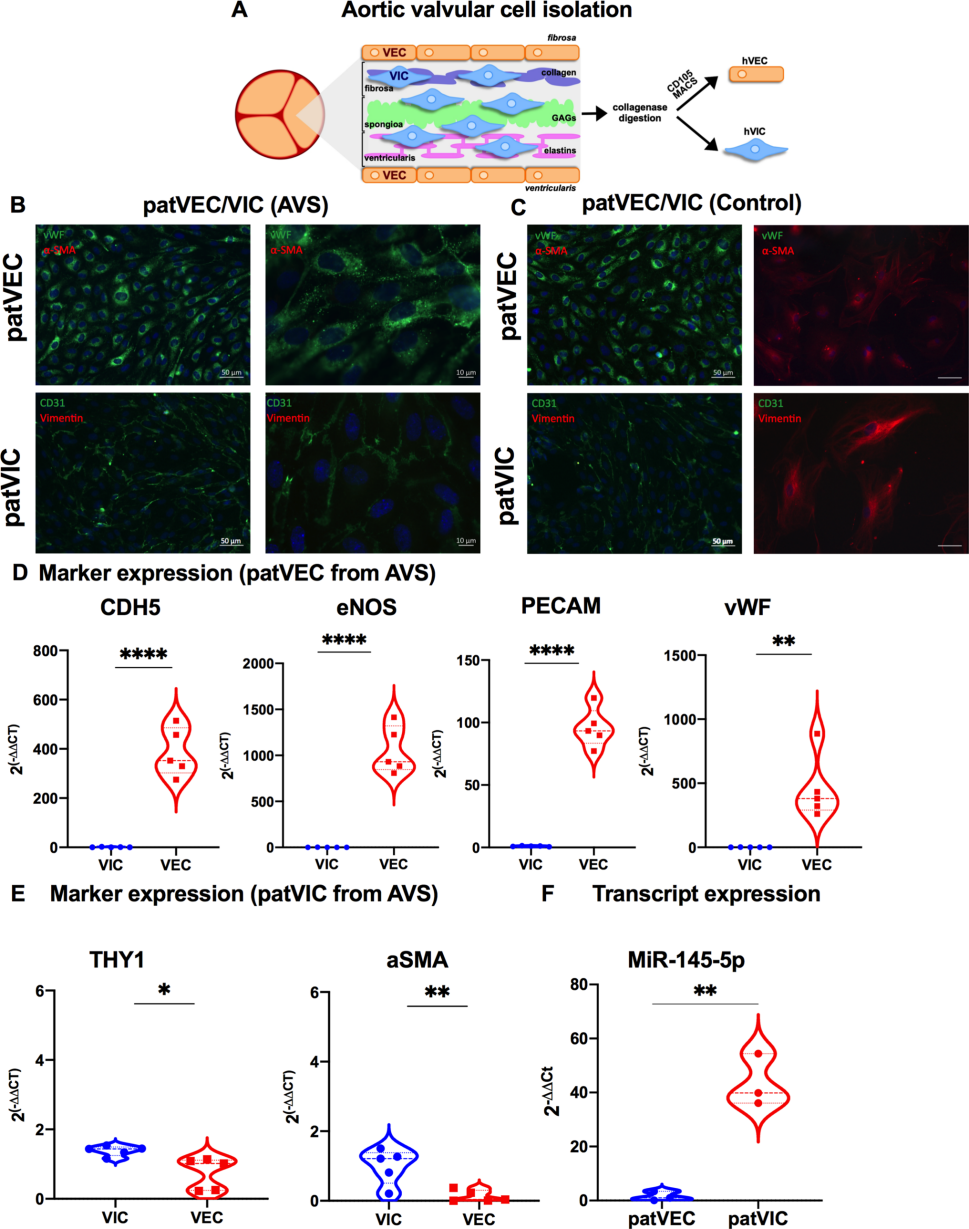
Isolation and characterization of patient-derived valvular cells. **A** Schematic representation of the structure of valvular matrix and isolation process of valvular cells from aortic valve tissue explant after SAVR. Minced aortic valve tissues have been subjected to collagenase digestions overnight at optimal cell culture conditions under gentle rotations. MACS affinity-based selection was performed to isolate VECs, whereas; a preplatig step is required for VICs to separate from the remaining cell mixture. *n* = 5 human calcified aortic valve (AVS) and *n* = 5, non-calcified (no AVS = Aortic insufficiency) aortic valves explanted from patients undergoing aortic valve replacement surgery were used to isolate valve cells (VIC and VEC). **B** Volcano plot showing differentially regulated human miRNAs in explanted valve tissues derived from patients who underwent SAVR. Thresholds (black dotted lines) of a two-fold change and p values (FDR-adjusted) < 0.05 were used to distinguish the miRNAs of interest. *n* = 4 for control (aortic insufficiency), *n* = 4 for aortic valve disease (AVS). **B**, **C** Characterization of isolated aortic valve cells using different surface markers, corresponding to VECs- and VICs-derived from AVS patients that underwent SAVR and compared with the corresponding controls. Representative immunofluorescence images of isolated VECs (patVECs) and VICs (patVICs) using their characteristic markers. **D**, **E** Expression of characteristic markers of isolated valvular cells, (VECs, and VICs), isolated from patients with AVS post-SAVR. Data are depicted as Student’s t tests. (****p < 0.0001, **p < 0.01, *n* = 5, two-tailed, unpaired). Expression of *miR-145-5p* was normalized to the internal control gene, *RNU6*. **F** Expression of *miR-145-5p* was measured in VECs and VICs isolated from patients with AVS after SAVR. Data are depicted as Student’s t test. (**p < 0.01, *n* = 3, two-tailed, unpaired). Expression of *miR-145-5p* was normalized to the internal control gene, *RNU6*. VICs, valvular interstitial cells; VECs, valvular endothelial cells; SAVR, surgical valve replacement; vWF, von Willebrand factor; a-SMA, alpha-smooth muscle actin; DAPI, 4′,6-Diamidin-2-phenylindol; PECAM1, platelet endothelial cell adhesion molecule; CDH5, cadherin 5/VE-Cadherin; NOS3, nitric oxide synthase 3/eNOS; DES, desmin; THY1, Thy-1 Cell Surface Antigen/CD90

To elucidate the mechanism of action of *miR-145-5p*, we developed several in vitro culture models. We established an improved isolation protocol for valvular endothelial cells (VECs) and valvular interstitial cells (VICs) from human calcified and non-calcified AV tissues explanted during SAVR (Fig. 3A). In brief, patient-derived VICs and VECs (patVIC and patVEC) were isolated from explanted aortic valves (AVs) through a multi-step collagenase digestion process, followed by Magnetic Activated Cell Sorting (MACS) using CD105 (Endoglin) to ensure endothelial cell purity (Fig. 3B). Additionally, we obtained human VICs and VECs (hVIC and hVEC) from a healthy young donor who passed away due to a non-cardiovascular-related cause. Both patient-derived and commercially sourced cells were characterized through qRT-PCR, assessing the expression of various endothelial and interstitial cell markers. A comparison of the expression of characteristic markers in these cells was performed (Figure S2A–D) to confirm the cellular identity of isolated and commercially sourced valve cells. Immunofluorescence staining for prototypical endothelial and interstitial cell markers (Fig. 3B, C, Figure S2A, B) and gene expression analysis by qRT-PCR (Fig. 3D, E, Figure S2C, D) were performed. Both patVICs and hVICs exhibited positive staining for α-SMA, which was further corroborated by qRT-PCR analysis (Fig. 3B–E). In contrast, patVECs and hVECs showed high expressions of endothelial markers, such as vWF, PECAM1, and CDH5 (Figure S2A–D), again confirming that our MACS-based isolation was valid and reproducible.

To investigate the cell-specific expression of *miR-145-5p* isolated from AV tissue after SAVR, we quantified *miR-145-5p* expression in VECs and VICs. Our qRT-PCR quantification revealed that VICs expressed a significantly higher level of *miR-145-5p* compared to VECs (Fig. 3F), suggesting that *miR-145-5p* may play a role in the pathogenesis of CAVD in mice and humans through its expression in specific valve cells.

### CAVD increases the level of miR-145-5p in aortic valve tissue and EVs derived from the plasma of patients

Recent studies have shown that various extracellular vesicles (EVs) (e.g., exosomes or small EVs, large EVs, apoptotic bodies) serve as vehicles for the transfer of short or long RNAs into nearby or distant recipient cells and play a role in cellular crosstalk [9, 16, 19]. Encapsulation of miRNAs in small or large EVs provides dramatic resistance to exonucleases found in blood or tissues. miRNAs can also be secreted bound to LDLs (low-density lipoproteins), HDL (high-density lipoproteins), and RBPs (e.g., Argonaute, hnRNPU, hnRNPUA2B1, hnRNPK, HuR) [8, 16, 19].

**Fig. 4:**
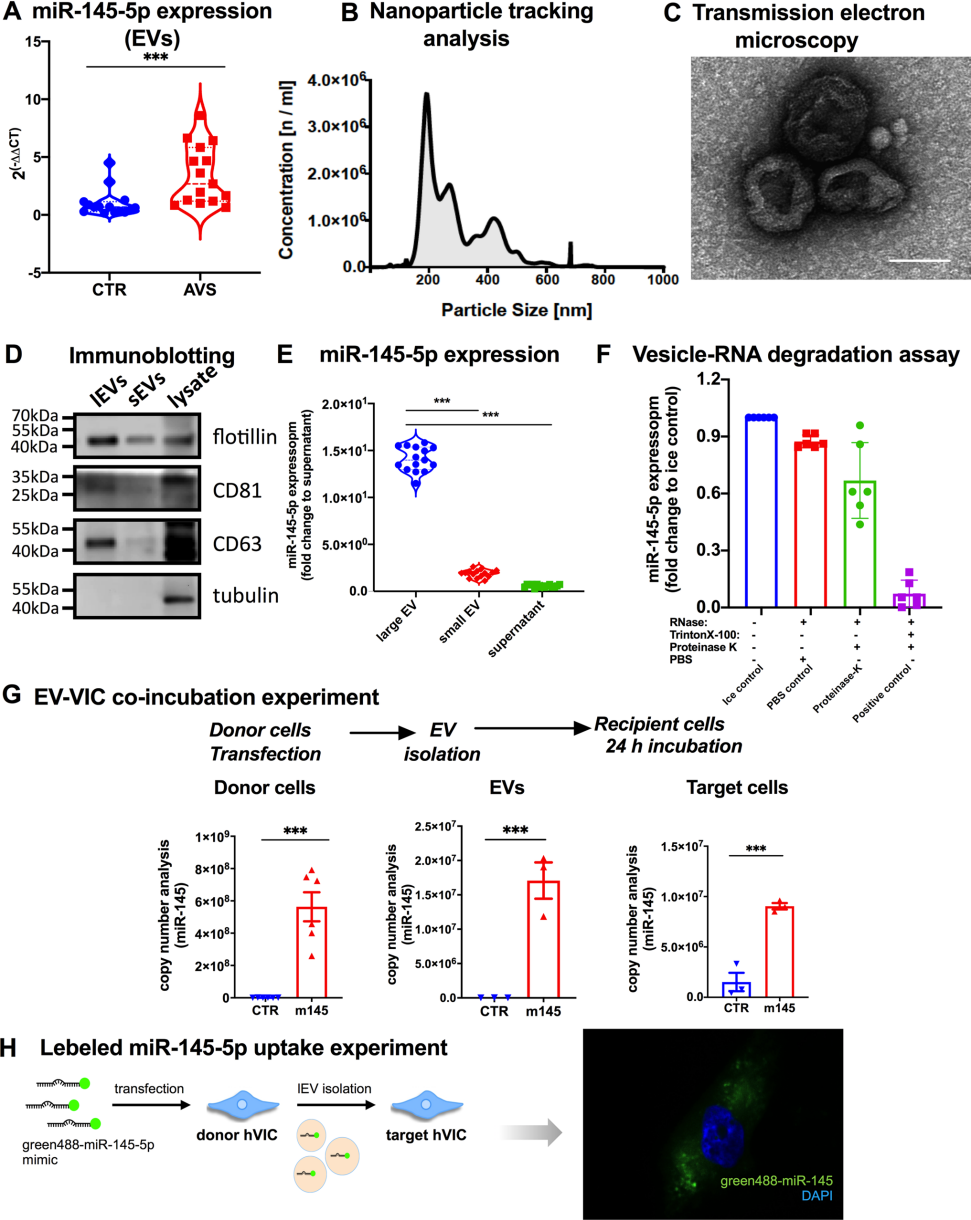
*MiR-145-5p* may regulate the calcification of recipient VICs via vesicular transfer. **A** Expression of *miR-145-5p* was measured in large EVs isolated from aortic valve tissues derived from patients with AVS and corresponding controls. Taqman-based expression analysis was performed to determine *miR-145-5p* in large EVs. Data are depicted with Student’s t tests. (***p < 0.001, *n* = 15, two-tailed, unpaired). Expression of *miR-145-5p* was normalized to the internal control gene, *RNU6*. Large EVs were isolated using centrifugation at 20,000 g according to the protocol published by our group and others (20–21, 29–31). **B** NTA was used to determine the diameter (~ 200–700 nm) and concentration of large EVs isolated from valve tissue from patients undergoing SAVR (**C**) TEM image (85,000 × magnification) of pelleted large EVs (diameter ~ 300–700 nm) derived from the valve tissue of patients with AVS. **D** Western blot analysis of the expression of small and large EV-markers. (E) *MiR-145-5p* expression was assessed in different EV populations isolated from VIC cultures by qRT-PCR (***p < 0.001, *n* = 15, by 1-way ANOVA with Bonferroni correction for multiple comparisons test). Cel-miR-39 was used for normalization. EVs and exosomes were isolated by centrifugation at 20,000 g and 100,000 g. **F** Vesicle–RNA degradation assays. VIC-derived EVs were treated in parallel using different conditions followed by RNase A digestion. *MiR-145-5p* was quantified by qRT-PCR (*p < 0.05, compared with the untreated group; ns: not significant, *n* = 3, by 1-way ANOVA with Bonferroni correction for multiple comparisons test). **G** Co-incubation experiments with large EV isolated from miR-145 overexpressed VIC and control. VICs were transfected with control, *miR-145-5p* mimic and large EVs were isolated and co-incubated with VICs under similar treatment conditions to quantify the differential *miR-145-5p* transfer via large EVs to the target cells. *MiR-145-5p* expression was assessed in donor VICs, isolated large EVs, and target VICs that were treated with large EVs, using copy number analysis (***p < 0.001, *n* = 6, by 1-way ANOVA with Bonferroni multiple comparisons test). **H** EV-incorporated *miR-145-5p* uptake experiments in VICs. Green fluorescent 488-labeled *miR-145-5p*-enriched EVs were isolated from donor VICs and co-incubated for 24 h to allow incorporation of EV-associated *miR-145-5p* into recipient VICs. EVs, extracellular vesicles; AVS, aortic valve stenosis; TEM, transmission electron microscopy; NTA, nanoparticle-tracking analysis; VECs, valvular endothelial cells; VICs, valvular interstitial cells

To identify the mode of *miR-145-5p* transport, we determined the plasma component of CAVD patients in which *miR-145-5p* can be detected. Following the isolation of large EVs, small vesicles (exosomes), and vesicle-free plasma (Figure S3A) [8, 9, 22, 23], the large EV population (170–800 nm) obtained from aortic valve tissues explanted from patients with aortic valve stenosis (AVS) after SAVR exhibited significantly higher miR-145-5p expressions compared to the control (vesicle-free plasma) (Fig. 4A). This finding suggests that miR-145-5p is primarily contained within large EVs. Characterization of EVs obtained from AV tissue or plasma was performed according to the current International Society of Extracellular Vesicles (ISEV) guidelines using nanoparticle-tracking analysis (NTA), transmission electron microscopy (TEM), and immunoblotting for vesicular markers, including tetraspanins (flotillin, CD63, CD81) (Fig. 4B–D).

To verify the secretion and transport of *miR-145-5p* in large EVs, a vesicle RNA degradation assay was performed (Fig. 4E, F). We observed that proteinase K digestion prior to RNase treatment had no impact on miR-145-5p levels. In contrast, pre-treatment with Triton X-100, a detergent that disrupts the phospholipid membrane of vesicles, resulted in nearly complete degradation of miR-145-5p upon subsequent RNase treatment. These findings suggest that extracellular miR-145-5p is incorporated into large EVs, shielding the RNA from degradation by resident or circulating nucleases. Collectively, the data indicate that extracellular miR-145-5p is primarily encapsulated and secreted within large EVs.

### Vesicular shuttling augments the expression level of miR-145-5p in recipient valvular interstitial cells

Given the pivotal role of VIC dysfunction in the development of AVS both in murine model and humans, we aimed to investigate whether VICs could transfer *miR-145-5p* to neighboring VICs. To this end, we established a co-culture model of donor and recipient VICs to explore intercellular communication via EVs. After transfection of VICs with *miR-145-5p* mimics, large EVs were isolated and incubated with target VICs. This revealed that *miR-145-5p* increased in target VICs in both control and mimic-transfected cells (Fig. 4G). This result suggests that horizontal EV-mediated cell-to-cell transfer of *miR-145-5p* increases *miR-145-5p* levels in target VICs.

To investigate whether the intercellular transfer of *miR-145-5p* via shuttling through large EVs occurs in a paracrine manner, fluorescently labeled large EVs were isolated from VICs and incubated with acceptor VICs. The results show that the fluorescently labeled EVs can be internalized into acceptor VICs (Figure S3B). Moreover, large EVs isolated from donor VICs transfected with fluorescently labeled *miR-145-5p* confirmed the uptake of EV-*miR-145-5p* into recipient VICs (Fig. 4H), suggesting that EV-mediated shuttling of *miR-145-5p* may act as an intercellular mediator of calcification within the aortic valve during stenosis progression.

### MiR-145-5p regulates pro-calcification marker genes in VICs

**Fig. 5:**
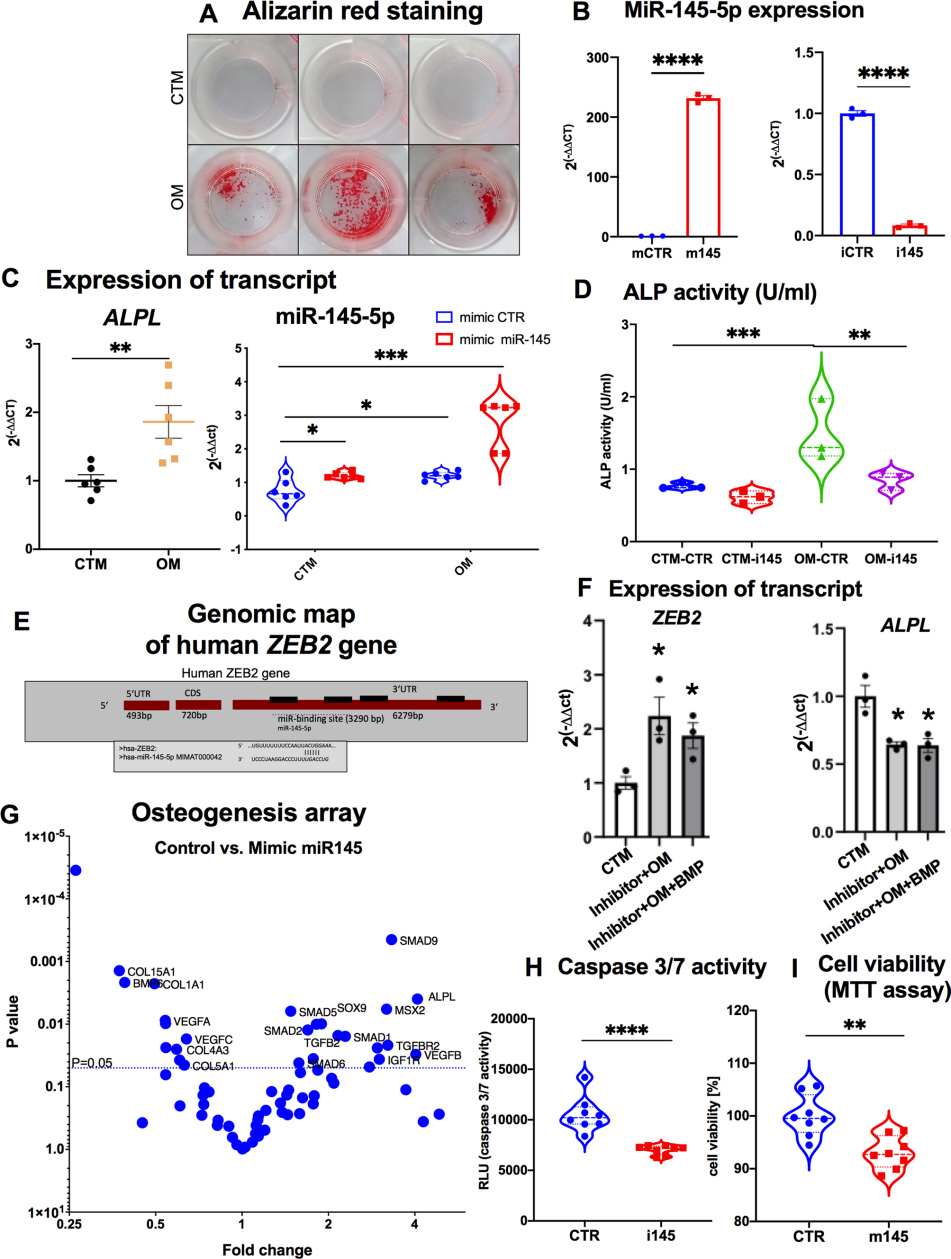
*MiR-145-5p* is a crucial regulator of valvular calcification and cellular function. **A** In vitro calcification experiment in VICs followed by quantification via Alizarin red staining on day 7. The deposition of calcium in the cellular matrix was stained and quantified with alizarin red after induction of in vitro calcification in VICs purchased from a commercial vendor (representative images of *n* = 3). **B** Overexpression and knockdown quantification of *miR-145-5p* by small oligonucleotides in VICs. Expression data for *miR-145-5p* in VICs have been compared with control and depicted as ****p < 0.001, *n* = 9, by 1-way ANOVA with Bonferroni multiple comparisons test. mCTR = mimic control, m145 = mimic miR-145-5p, iCTR = inhibitor control, i145 = inhibitor miR-145-5p. **C** Taqman-based quantification of gene expression of ALPL as a hallmark of calcification of VICs and *miR-145-5p* after induction of in vitro calcification using osteogenic medium (OM) for 7 days. Expression data for ALPL mRNA in VICs and *miR-145-5p* has been compared with control medium and control *miR-145-5p* mimic and depicted as*p < 0.05, **p < 0.01, ***p < 0.001, *n* = 6, by 1-way ANOVA with Bonferroni multiple comparisons test. **D** Quantification of ALP activity in VICs after transient *miR-145-5p* inhibition and simultaneous incubation with OM (*n* = 3, 2-way ANOVA with Bonferroni multiple comparisons test). **E** Genomic map of the ZEB2 gene, a known regulator of ALPL, with predicted binding sites for hsa-*miR-145-5p*, in the 3’-UTR. ZEB2 is a known repressor of ALPL and contains multiple binding sites for numerous miRs, including, miR-145-5p. **F** Expression of ZEB2 and ALPL mRNA in VICs after induction of in vitro calcification for 7 days. Data is depicted as *p < 0.05, *n* = 3, by 1-way ANOVA with Bonferroni multiple comparisons test. **G** RT^2^ Profiler PCR array analyses (Osteogenesis) were performed on VICs upon miR-145 overexpression and control (*n* = 5). Volcano plot shows the differentially regulated genes between miR-145-5p overexpression and control VICs by PCR array. The downregulated genes with a > two-fold change *p* < 005 were labeled and highlighted. **H** The level of apoptosis was assessed via caspase 3/7 activity after incubation with H_2_O_2_ (100 μM) for 24 h. The data was analyzed by Student’s *t* test, 2-tailed, unpaired. (****p < 0.001, *n* = 8). **I** Cell viability was determined using MTT assays (incubation with H_2_O_2_ (100 μM)). Data were analyzed by Student’s t test, 2-tailed, unpaired. (**p < 0.01, *n* = 8). ZEB2, Zinc Finger E-Box Binding Homeobox 2; ALPL, Alkaline Phosphatase; CTM, control media, OM, osteogenic medium; UTR, untranslated region; VICs, valvular interstitial cells; miRs, microRNAs; H_2_O_2_, hydrogen peroxide; AVS, aortic valve stenosis

Recent studies have reported that miRNAs can influence the calcification process in vascular and heart valve cells via the regulation of pro-calcifying genes or proteins [12, 24]. To investigate whether calcification of VICs is mediated by *miR-145-5p*, we performed in vitro calcification experiments. The extent of calcification was determined using alizarin red staining to quantify deposition of calcium within the matrix (Fig. 5A). To further clarify a direct involvement of *miR-145-5p* in calcification of VICs, we examined the expression of calcification marker genes by qRT-PCR in cells treated with either a *miR-145-5p* mimic or an inhibitor (Fig. 5B, Figure S4A-B). Upon silencing of miR-145-5p, an interesting observation was the downregulation of several calcification-related genes. These included alkaline phosphatase (ALPL), zinc finger E-box binding homeobox 2 (ZEB2), RUNX family transcription factor 2 (RUNX2), secreted phosphoprotein 1 (SPP1), matrix Gla protein (MGP), KLF transcription factor 4 (KLF4), and SMAD family member 5 (SMAD5), which are all associated with biomineralization. These data suggest that *miR-145-5p* may regulate key genes in calcification processes that can be partially abrogated via overexpression of *miR-145-5p* (Figure S4A-B).

Among the dysregulated genes involved in calcification processes, ALPL was observed to be upregulated in the induction of calcification in *miR-145-5p* overexpressed VICs (Figure S4C). The correlation between *miR-145-5p* and ALPL was further confirmed when VICs were incubated with osteogenic medium (OM) to induce calcification (Fig. 5C), suggesting that *miR-145-5p* regulates ALPL expressions in a dose-dependent manner (Figure S4C). To investigate whether *miR-145-5p* regulates ALPL transcription or ALP activity itself, we quantified alkaline phosphatase activity after transfection of VICs with mimics and inhibitors and subsequent induction of calcification in vitro. After knockdown of *miR-145-5p* in VICs, we observed decreased enzymatic activity of ALP (Fig. 5D), suggesting that *miR-145-5p* can modulate the expression of ALPL and thus decrease ALP activity. To investigate whether *miR-145-5p* directly controls the function of VICs related to the pathogenesis of CAVD, we performed a series of experiments focusing on migration by scratch wound assays (Figure S4D), proliferation rate by BrdU incorporation (Figure S4E), and apoptosis by caspase 3/7 activity (Figure S4F) in VICs. Altogether, data demonstrated that miR-145-5p reduces cellular migration and apoptosis. Based on the experimental data, this suggests that *miR-145-5p* is a crucial regulator of VIC function associated with CAVD.

### MiR-145-5p may regulate calcification of VICs through ZEB2 and tissue nonspecific alkaline phosphatase (ALPL)

Several studies revealed that *ALPL* transcription can be regulated by numerous transcription factors (TFs). For example, ZEB2 is a known repressor of the *ALPL* and was reported to be increased in healthy heart tissues, including AV tissue [7, 10, 17]. Given that *miR-145-5p* was reported to bind to *ZEB2* to regulate cellular gene expression during epithelial–mesenchymal transition (EMT) in metastasis [25], we sought to further characterize its regulation by *miR-145-5p*. To study whether *ZEB2* has binding sites for *miR-145-5p,* we analyzed the genomic architecture of the *ZEB2*. Genomic analysis and target prediction of *miR-145-5p* binding demonstrates that the *ZEB2* contains potential binding sites for numerous miRNAs, including *miR-145-5p*, 3290 bp upstream of the first two exons in its 3’-UTR (untranslated region). This suggests that *miR-145-5p* may bind to *ZEB2* (Fig. 5E), consequently regulating the expression of its cognate *ALPL* mRNA and thus protein synthesis.

Given that ALPL is a central player in calcification [7, 9, 10], we sought to further characterize its regulation by *miR-145-5p*. We reciprocally quantified the expression of *ALPL* and its transcriptional repressor*, ZEB2*, in the same experiments upon knockdown of *miR-145-5p*. The induction of calcification by osteogenic medium showed that the expression levels of *ZEB2* and *ALPL* are inversely correlated, an effect also seen upon induction of *ZEB2* by BMP2 (bone morphogenic proteins 2) (Fig. 5F), suggesting that *miR-145-5p* regulates the transcription of the *ALPL* gene via its transcriptional repressor *ZEB2* by binding to the 3’-UTR of *ZEB2*. To provide another layer of evidence that ALPL is directly regulated by miR-145-5p-ZEB2 axis, we performed unbiased osteogenesis array containing important genes that regulates genes in valvular and vascular calcification of cells (Fig. 5G). Our data demonstrated that crucial regulators of osteogenesis pathways were significantly changed (e.g., *SMAD 2, 5, 9, TGFB2, SOX9,* and *ALPL,* etc.). To examine whether this regulation has any effect on the cellular function of VICs, we performed experiments focusing on cellular viability and functions, namely activation of caspase 3/7 for apoptosis, cell viability via MTT reduction, and cell migration via scratch wound healing assays upon silencing or overexpressing *miR-145-5p.* The results revealed that *miR-145-5p* can regulate apoptosis and viability of VICs as a pro-apoptotic miRNA (Fig. 5G–I). Taken together, our data suggest that *miR-145-5p* exerts a pro-apoptotic effect that is a prerequisite for the initiation of valvular calcification in CAVD.

### *RNA sequencing reveals that *in vitro* calcification alters calcific gene and miRNA expression profiles in valvular interstitial cells*

**Fig. 6:**
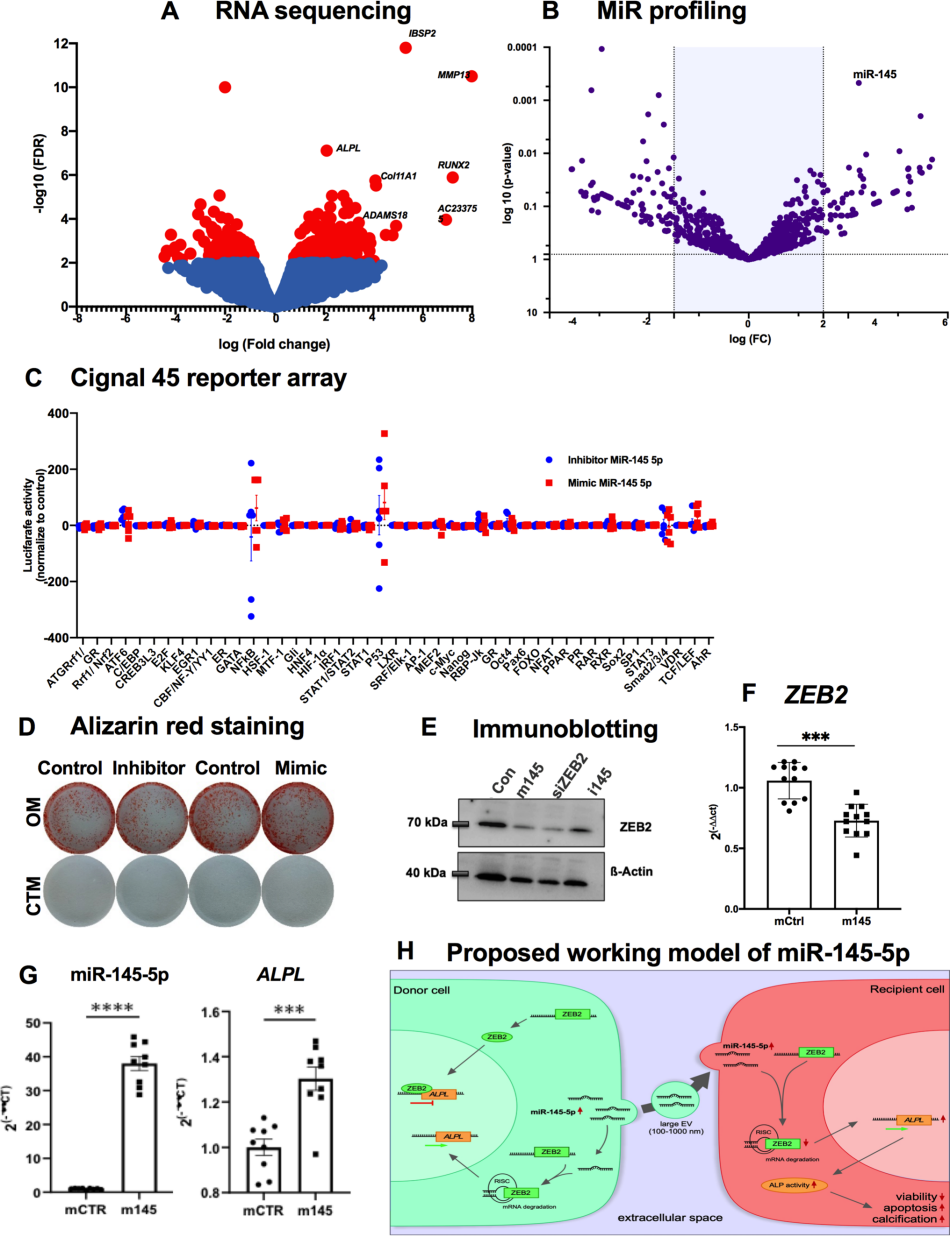
Transcriptomic profiling of calcified VICs confirms that miR-145-5p regulates calcification and apoptosis by regulating *ALPL2* and other calcific genes. **A** Volcano plot of RNA sequencing data of calcified human VICs (7 days) vs. controls that demonstrated an upregulation of genes that are central to calcification, such as *ALPL, RUNX2, MMP13, ADAMTS18, COL11A1,* and *AC233755,* etc. Furthermore, an upregulation of known inflammation-, and apoptosis-regulated genes upon induction of calcification for 7 days was observed (*n* = 4; SEM; FDR-adjusted). **B** Volcano plot with profiling dysregulated miRNAs in calcified VICs (7 days) in comparison to control VICs with a cut-off 1.5 fold (*n* = 4; SEM; FDR-adjusted). The top and statistically significantly upregulated miRNAs are represented. (**C**) A high-throughput transcription factor activity reporter luciferase assay upon oligonucleotide-mediated depletion or overexpression of miR-145 vs. control in VICs was performed 48 h after transfection. Depicted are the activities of 45 transcription factors in inhibitor, *miR-145-5p*-treated cells and control cells (control-treated) 48 h after transfection with the reporter constructs. **D** Representative bright field images of in vitro calcification experiment in VICs followed by quantification via Alizarin red staining on day 7. The deposition of calcium in the cellular matrix-quantified and stained with alizarin red after induction of in vitro calcification in VICs purchased from a commercial vendor (representative images of *n* = 3). **E** Western blotting for ZEB2 protein after transfection with control, *miR-145-5p* inhibitor (Inhibitor), siRNA against ZEB2 (siZEB2) and *miR-145-5p* mimic (Mimic). **F** Overexpression data of *ZEB2* mRNA in VICs upon transfection and subsequent induction of calcification for 7 days to assess the expression of the transcripts (^n^***p < 0.001, *n* = 12, by Student’s t test, unpaired). **G** Overexpression data of *miR-145-5p*, and *ALPL* in VICs upon transfection and subsequent induction of calcification for 7 days to assess the expression of the transcripts (^ns^*p* > 0.05, ***p < 0.001, ****p < 0.0001, *n* = 9, by 1-way ANOVA with Bonferroni multiple comparisons test). **H** Large extracellular vesicular *miR-145-5p* regulates calcification and apoptosis of valvular interstitial cells by regulating *ZEB2-ALPL axis* and other apoptotic genes. The model proposed for *miR-145-5p* action in which it is shuttled to the recipient cells via ZEB2–*miR-145-5p* conjugates to regulate calcific gene networks by binding to 3'-UTR, such as ALPL, to control the inflammation, calcification, and apoptosis of VICs. ICs, valvular interstitial cells; miRs, microRNAs; RISK, RNA-induced silencing complex; UTR, untranslated region; ZEB2, Zinc Finger E-Box Binding Homeobox 2; RUNX2, Runt-related transcription factor 2; ALPL, Alkaline Phosphatase; HSP27, Heat shock protein 27; HIF1a, Hypoxia-inducible factor 1-alpha; CTM, control media, OM, osteogenic medium; UTR, untranslated region; VICs, valvular interstitial cells; miRs, microRNAs; AVS, aortic valve stenosis

To assess calcification-induced gene expression signatures in the VICs, we performed bulk RNA sequencing of calcified VICs upon incubation with OM for 7 days and controls (without OM), resulting in a robust differential expression of pro-calcific markers, e.g., *ADAMS18, RUNX2, ALPL, COL11A1,* and *MMP13* (Fig. 6A). We further analyzed the miRNA expression profile of these VICs. We found *miR-145-5p* among the dysregulated miRs (Fig. 6B), providing further confirmation of our in vitro and in vivo data, which identify *miR-145-5p* to be involved in the calcification process of VICs. Further bioinformatics analysis via DAVID (Database for Annotation, Visualization and Integrated Discovery) and KEGG (Kyoto Encyclopedia of Genes and Genomes) revealed that induced pathways involve osteogenesis and apoptosis among the top-regulated pathways, providing further evidence of the calcification potential of VICs (Figure S5A).

### *MiR-145-5p may exert its function *via* the miR-145-5p-ZEB2-ALPL axis in calcific valvular interstitial cells*

To gain further insights into the cellular pathways related to *miR-145-5p*-regulated calcification, an unbiased high-throughput reporter array for transcription factor activity was performed. Among the highly active transcription factors, *NFkB (Nuclear Factor Kappa B Subunit 1), STAT1 (Signal Transducer And Activator Of Transcription 1), STAT3 (Signal Transducer And Activator Of Transcription 3), *and* P53 (cellular tumor antigen p53)* were dysregulated in the reporter array analysis (Fig. 6C, Figure S5B, Table S1).

Next, we investigated whether overexpression or knockdown of *miR-145-5p* supports our data and whether overexpression of *miR-145-5p* abrogates the pro-apoptotic/calcifying effect of ZEB2 and ALPL. We repeated calcification in VICs after overexpression and knockdown of *miR-145-5p* followed by alizarin red staining, and found that overexpression of miR-145-5p directly increased calcification (Fig. 6D). As anticipated, the quantification of ZEB2 protein expression through immunoblotting in VICs treated with the miR-145-5p mimic and siRNA targeting ZEB2 revealed a reduced expression of ZEB2 in both mimic- and siZEB2-treated VICs. In contrast, ZEB2 expression was elevated in inhibitor-treated VICs. These results indicate that miR-145-5p directly regulates ZEB2, which functions as a transcriptional repressor of ALPL expression (Fig. 6E). To determine the involvement of *miR-145-5p-ZEB2*-ALPL as a mediator of the calcification process in VICs, we further quantified mRNA expression of *ZEB2*, which supports our finding that overexpression of *miR-145-5p* represses *ZEB2*, thereby increasing the expression of ALPL in VICs (Fig. 6F).

Taken together, our results indicate that regulation of *ZEB2-ALPL* by *miR-145-5p* triggers apoptosis and calcification of VICs and that the level of *miR-145-5p* expression is important for this mechanism, which acts in a *miR-145-5p-ZEB2-ALPL*-dependent manner (Fig. 6G, H). Overexpression of *miR-145-5p* in the aortic valve leads to inflammation, calcification, and apoptosis, which may result in stiffening of the aortic valve cusps and narrowing of the aortic valve orifice, ultimately leading to left ventricular pressure overload and heart failure.

## Discussion

The data of the present study show a newly identified role for EV-miRNAs, which are differentially expressed in CAVD patients and are associated with calcification. *Mir-145-5p* expression correlates with calcification of AV tissues explanted from patients undergoing SAVR for severe CAVD and was found to have predictive value for calcification in CAVD. Based on the clinical results of our study, we further characterized and investigated the functional role of *miR-145-5p* in heart valvular interstitial cells (VICs) in clinical and pathological mouse models. It was found that the concentration of *miR-145-5p* in VICs isolated from aortic valve tissue explants of aortic stenosis patients/CAVD mice was higher than in control patients/sham-operated mice and was released into the circulation in a vesicle-associated form. Mechanistically, *miR-145-5p* interferes with translation of *ZEB2* mRNA by binding to its 3’-UTR. Since it is a negative regulator of the *ALPL* gene, a reduction of *ZEB2* leads to an upregulation of *ALPL* expression and thus to a calcification of the VICs. The effects induced by *miR-145-5p* in vitro, such as reduction of viability and migration, enhancement of inflammation, calcification and simultaneous induction of apoptosis, are considered to be prerequisites for the initiation of the pathogenesis of CAVD.

EV-mediated cell–cell crosstalk is gaining increased interest in the scientific community due to its importance for non-invasive diagnostics and therapeutic potential. Circulating EVs can be used as biomarkers for disease as has been shown in various cancers and also in cardiovascular diseases, such as atherosclerosis and vascular calcification [8, 12, 13, 39, 41]. EVs can be secreted by a variety of cells relevant to cardiovascular disease, such as ECs, platelets, and immune cells. Higher concentrations of circulating EVs are observed in patients with classic cardiovascular risk factors, such as smoking, hypertension, diabetes mellitus, and dyslipidemia, as well as in patients with coronary artery disease (CAD) [16]. Elevated concentrations of circulating endothelial cell-derived EVs correlate with higher rates of major adverse cardiovascular and cerebral events (MACCE) in patients with stable CAD [6, 13, 14, 16, 19]. Not only the EV concentration but also the composition of its cargo can predict and influence cardiovascular events. One of the first studies to demonstrate this showed that higher concentrations of EV-bound miR-126 and miR-199a were inversely correlated with MACCE and revascularization-free survival in a cohort of patients with stable CAD [19]. These observations prompted several mechanistic studies demonstrating the positive and negative effects of EVs and their cargo on EC and smooth muscle cells (SMC) during the course of atherosclerosis. MiRNAs transferred into EVs are involved in endothelial regeneration, SMC phenotype switching, osteoblastic differentiation and vascular calcification [21]. Under calcification conditions, EVs can be packaged with miRNAs targeting mRNAs for proteins actively involved in osteoblastic differentiation and calcification by a variety of cell types, including SMCs, ECs, and macrophages, demonstrating their important role in vascular calcification processes [1, 2, 12–14, 19, 20]. Recently, we demonstrated that EV-incorporated *miR-122-5p* post-transcriptionally represses *BCL2*, an anti-apoptotic gene, which is central to cell viability and apoptosis. The levels of circulating EV-bound *miR-122-5p* were found to be an indicator of heart function in patients with low or no LVEF improvement after TAVR [14]. EV-incorporated miRNAs have the potential to bind mRNAs that encode for master regulators of osteoblastic differentiation and calcification, such as RUNX2 *(runt-related transcription factor 2)* and ALPL *(alkaline phosphatase)*. However, the involvement of EV-bound miRNA cargoes in valvular calcification remains largely unexplored.

*MiR-145-5p* belongs to the *miR-143/145* cluster of miRNAs, which has been reported to be dysregulated (either as a cluster or one of its members) in essential hypertension, atherosclerosis, CHD and pulmonary arterial hypertension (PAH) [5, 11, 13]. Patients with stable CAD have lower concentrations of circulating *miR-145-5p* than healthy controls, but patients with unstable angina show increased concentrations, and miR-145-5p concentrations correlate with infarct size in myocardial infarction. Moreover, *miR-145-5p* can be transferred between endothelial and vascular SMCs via EVs [11]. Physiologic laminar flow induces expressions of *miR-145-5p* in ECs in a KLF2-dependent manner, leading to packaging in sEVs and transfer to vascular SMCs, where *miR-145-5p* induces an atheroprotective vascular SMC phenotype [11]. During myocardial infarction, *miR-145-5p* also appears to exert a protective effect by inhibiting apoptosis via the Akt3/mTOR signaling pathway [32, 37–39]. In contrast, the *miR-143/145* cluster was found to be upregulated in symptomatic atherosclerotic carotid plaques compared to asymptomatic controls, suggesting a role in plaque destabilization [32]. On the other hand, overexpression of *miR-145-5p* in ApoE-/- mice using lentiviral vectors was able to reduce the size of aortic atherosclerotic plaques. These stabilized plaques exhibited a thicker fibrous cap, higher collagen content and fewer pro-inflammatory macrophages than plaques from untreated littermates [32]. In PAH patients, *miR-143* and *miR-145* levels were found to be higher in pulmonary arterial SMCs (PASMCs) than in healthy controls. In vitro experiments showed that *miR-145-5p* affects migration and apoptosis of PASMCs while knockdown of *miR-145-5p* ameliorated the development of PAH in a hypoxia-induced PAH mouse model in vivo [4, 21, 30]. Recently, *miR-143* was shown to promote heart valve calcification by inhibiting matrix Gla protein (MGP), which in turn inhibits calcification and is necessary for heart valve homeostasis [8]. Thus, the *miR-143/145* cluster appears to have multiple functions in the cardiovascular system, ranging from protective to detrimental.

*MiR-145-5p* had not previously been investigated in the pathology of CAVD. To further validate the involvement of this miRNA in CAVD, we examined the AV of mice that had undergone wire-induced injury of the CAVD and developed severe CAVD. In line with the patient data, we observed upregulation of *miR-145-5p* in the AV tissue of diseased mice. Since our initial screenings were performed on whole human tissue samples, we sought to identify the cell type that might be responsible for this increase. MiRNA analysis of primary cells from human tissue explants demonstrated a significantly higher expression of *miR-145-5p* in VICs than VECs, suggesting a role during calcification and osteoblastic differentiation. To investigate the function of *miR-145-5p* in VICs, we analyzed its expression in vitro under calcifying conditions. A so-called osteogenic medium (OM) induces calcification and osteoblastic differentiation of VICs in an ALPL-dependent manner. In our in vitro experiments, *miR-145-5p* and ALPL levels were significantly increased under calcifying stimuli. In silico target prediction revealed a specific *miR-145-5p*-binding site in the 3’UTR region of ZEB2/SIP1. Of note, the binding of *miR-145-5p* to this binding site in ZEB2 has already been confirmed by a luciferase promotor assay in another experimental setting [42]. Importantly, ZEB2 has been shown to be a transcriptional repressor of *ALPL* [42]. The qRT-PCR analysis confirmed that ZEB2 expression decreases under calcifying conditions and negatively correlates with *miR-145-5p* and ALPL mRNA levels in vitro. To further elucidate the *miR-145-5p*/ZEB2/ALPL axis, we overexpressed *miR-145-5p* in VICs, which led to a significant downregulation of ZEB2 and a consecutive upregulation of ALPL. Furthermore, cells that were simultaneously treated with OM and *miR-145-5p* mimic showed an increased ALPL expression, while *miR-145-5p* ablation diminished ALPL in OM incubated cells, thus indicating an important role of *miR-145-5p* in the modulation of ALPL-dependent calcification.

Since we observed higher levels of *miR-145-5p* in AV tissue- and blood-derived large EVs (IEVs) from CAVD patients, we sought to further investigate the transfer of *miR-145-5p* between VICs via EVs. Fluorescence microscopy and copy number experiments confirmed the uptake of PKH26-labeled EVs (a lipophilic membrane dye), fluorescently labeled-*miR-145-5p*, and unlabeled *miR-145-5p* containing EVs by target cells, demonstrating intercellular communication between VICs via EV-bound *miR-145-5p.* The transfer of miRNA cargo between valvular cells might therefore play an important role in CAVD initiation and progression. The herein identified regulatory *miR-145-5p-*ZEB2-ALPL axis presents a potential target for the development of new, RNA-based therapies. Furthermore, in vivo experiments utilizing EV-incorporated *miR-145-5p* mimics and inhibitors will lead to a better understanding of the involved mechanisms and suggest possible therapeutic strategies. Taken together, in this study, using unbiased miRNA profiling, we have identified significantly dysregulated miRNAs in AV tissue from patients with CAVD. Among several miRNAs that are commonly dysregulated in mice and humans under CAVD, *miR-145-5p* levels were most significantly upregulated and selected for further validation in a larger patient cohort, which subsequently confirmed its expression signature in CAVD patients. *MiR-145-5p* levels in tissue-derived and circulating large EVs isolated from CAVD patients were also higher when compared to control (no CAVD) patients. By RNA sequencing and high-throughput TF arrays utilizing our in vitro calcification model of VICs, we revealed that *miR-145-5p* regulates a key process in calcification, i.e., inhibition of ZEB2, a DNA-binding transcription factor that regulates transcription and translation of the ALPL protein, regarded as a hallmark of calcification in valvular and vascular calcification.

In clinical cardiology, identification of patients at risk to develop CAVD is a persistent problem. Our studies have revealed that expression levels of EV-ncRNAs are associated with a risk of developing valvular calcification and CAVD. However, it’s still vastly challenging whether EV-ncRNA expression can i) selectively identify patients at risk, ii) diagnose the prevalence and severity of early/severe CAVD, iii) monitor CAVD disease progression, and iv) predict a patient’s individual outcome with a long-term follow-up. These conclusions from in vitro experiments are based on transient overexpression or inhibition of *miR-145-5p* and therefore do not allow us to firmly conclude that *miR-145-5p* is an indispensable factor to promote direct calcification processes in the aortic valve. Importantly, *miR-145-5p* is highly overexpressed in mice and human calcified AV tissues and silencing of *miR-145-5p* reduces apoptosis of VICs and promotes migration and viability of VICs, suggesting that the potential therapeutic effects of pharmacological *miR-145-5p* inhibition for CAVD treatment/prevention may be transferable into human CAVD patients.

### Clinical significance

#### What is known?

- Calcific aortic valve disease (CAVD) is the most prevalent structural heart valve disease requiring surgical or interventional valve replacement. Currently, there is no medical treatment option that can slow, halt, or reverse the progression of the disease.
- CAVD leads to pressure overload of the left ventricle (LV), resulting in concentric hypertrophy and LV dysfunction.
- CAVD is not an exclusively degenerative disease leading to fibrosis and calcification of the valve cusps, but rather a chronic inflammatory disease in which mechanical stress and shear stress lead to endothelial dysfunction and immune cell infiltration, resulting in chronic inflammation, apoptosis and differentiation of the interstitial cells of the heart valves into osteoblast-like cells.
- Increasing osteoblastic differentiation and the formation of macrocalcifications are hallmarks of the later stages of CAVD.

#### What is the new information we provide?

- During CAVD, the expression pattern of tissue-derived and vesicle-enriched regulatory miRNAs changes.
- Patient-derived aortic valve tissue as well as in aortic valve explants from an experimental murine aortic valve stenosis model demonstrated an increased expression of *miR-145-5p* in humans and mice.
- *MiR145-5p* contributes to calcification of the aortic valve through ZEB2, a transcriptional repressor of ALPL, in valvular interstitial cells.
- Extracellular vesicle shuttling of *miR-145-5p* contributes to valvular cell–cell crosstalk and plays a role in the pathogenesis of CAVD.

## Supplementary Information

Below is the link to the electronic supplementary material.

Supplementary file1 (DOCX 6754 KB)

Supplementary file2 (XLSX 127 KB)

## Data Availability

All data and materials are available upon contact with corresponding author.

## Abbreviations

CAVD: Calcific aortic valve disease
CVD: Cardiovascular disease
ECs: Endothelial cells
EVs: Extracellular vesicles
VICs: Valvular interstitial cells
VECs: Valvular endothelial cells
HCAEC: Human coronary artery endothelial cell
LV: Left ventricle
LVEF: Left ventricular ejection fraction
miR: MicroRNA
miRNA: MicroRNA
NGS: Next-generation sequencing
RBP: RNA-binding protein
RIP: RNA immunoprecipitation
SAVR: Surgical aortic valve replacement
TAVR: Transcatheter aortic valve replacement
TEM: Transmission electron microscopy
